# The Potential Role of Sildenafil in Cancer Management through EPR Augmentation

**DOI:** 10.3390/jpm11060585

**Published:** 2021-06-21

**Authors:** Mohamed Haider, Amr Elsherbeny, Valeria Pittalà, Antonino N. Fallica, Maha Ali Alghamdi, Khaled Greish

**Affiliations:** 1 Department of Pharmaceutics and Pharmaceutical Technology, College of Pharmacy, University of Sharjah, Sharjah 27272, United Arab Emirates; 2 Research Institute of Medical & Health Sciences, University of Sharjah, Sharjah 27272, United Arab Emirates; 3 Division of Molecular Therapeutics and Formulation, School of Pharmacy, University of Nottingham, Nottingham NG7 2RD, UK; amr.elsherbeny@nottingham.ac.uk; 4 Department of Drug and Health Science, University of Catania, 95125 Catania, Italy; vpittala@unict.it (V.P.); antonio.fallica93@gmail.com (A.N.F.); 5 Department of Biotechnology, College of Science, Taif University, Taif 21974, Saudi Arabia; mahaasg@agu.edu.bh; 6 Department of Molecular Medicine, Princess Al-Jawhara Centre for Molecular Medicine, School of Medicine and Medical Sciences Arabian Gulf University, Manama 329, Bahrain

**Keywords:** sildenafil, phosphodiesterase 5 inhibitors, drug repurposing, cancer, chemoadjuvant

## Abstract

Enhanced permeation retention (EPR) was a significant milestone discovery by Maeda et al. paving the path for the emerging field of nanomedicine to become a powerful tool in the fight against cancer. Sildenafil is a potent inhibitor of phosphodiesterase 5 (PDE-5) used for the treatment of erectile dysfunction (ED) through the relaxation of smooth muscles and the modulation of vascular endothelial permeability. Overexpression of PDE-5 has been reported in lung, colon, metastatic breast cancers, and bladder squamous carcinoma. Moreover, sildenafil has been reported to increase the sensitivity of tumor cells of different origins to the cytotoxic effect of chemotherapeutic agents with augmented apoptosis mediated through inducing the downregulation of Bcl-xL and FAP-1 expression, enhancing reactive oxygen species (ROS) generation, phosphorylating BAD and Bcl-2, upregulating caspase-3,8,9 activities, and blocking cells at G0/G1 cell cycle phase. Sildenafil has also demonstrated inhibitory effects on the efflux activity of ATP-binding cassette (ABC) transporters such as ABCC4, ABCC5, ABCB1, and ABCG2, ultimately reversing multidrug resistance. Accordingly, there has been a growing interest in using sildenafil as monotherapy or chemoadjuvant in EPR augmentation and management of different types of cancer. In this review, we critically examine the basic molecular mechanism of sildenafil related to cancer biology and discuss the overall potential of sildenafil in enhancing EPR-based anticancer drug delivery, pointing to the outcomes of the most important related preclinical and clinical studies.

## 1. Introduction 

**Figure 1:**
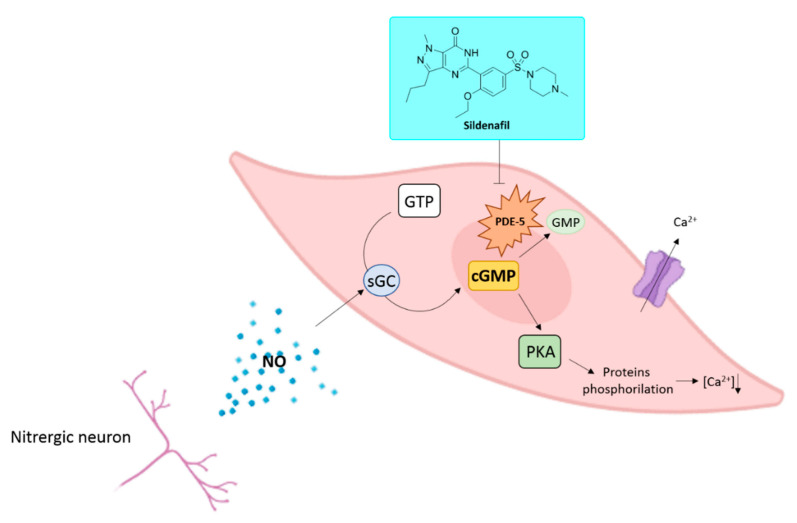
NO/sGC/cGMP pathway and sildenafil mechanism of action in erectile tissues and chemical structure of sildenafil.

Sildenafil, (5-(2-ethoxy-5-((4-methylpiperazine-1-yl)sulfonyl)phenyl)-1-methyl-3-propyl-1H-pyrazolo[3,4]-d]pyrimidin-7(6H)-one), sold as citrate salt, is a drug primarily prescribed for the treatment of ED (Figure 1). Sildenafil exerts its biological effects through the inhibition of PDE-5 [1,2]. Phosphodiesterases are a class of enzymes responsible for the degradation of cyclic AMP (cAMP) or GMP (cGMP) to their respective nucleotides 5′-AMP and 5′-GMP.

Nowadays, 11 PDE isoforms have been identified [3]. These isozymes share an aminoacidic homology superior to 65% and differ for their tissue distribution and affinity toward cAMP or cGMP; the latter is specifically degraded by PDE-5, -6, and -9 [4,5,6]. PDEs exert their catalytic activity as homodimers [7,8]. In each monomer, it is possible to highlight the presence of a zinc-binding motif, a catalytic binding pocket, two allosteric sites able to bind cAMP or cGMP, and a residue of serine in position 92 whose phosphorylation enhances the enzymatic activity through the activation of protein kinases A and G (PKA and PKG) [7,9]. PDEs regulate in an isoform-dependent manner different physiological roles such as platelet aggregation, inflammation, immune system activation, hormone secretion, vision, cardiac contractility, and muscle metabolism, smooth muscle contractility, depression, calcium intracellular concentration, cell proliferation, and penile erection [10]. The latter is an event that originates from the release of the gasotransmitter nitric oxide (NO) by nitrergic neurons and endothelial cells in case of sexual stimulation [11]. The physicochemical properties of NO allow it to diffuse into cells activating the enzyme soluble guanylyl cyclase (sGC) that in turn converts GTP into cGMP. In erectile tissues, cGMP triggers the phosphorylation of specific proteins involved in the modulation of the intracellular calcium ions concentration. A decreased concentration of calcium ions through the activation of Ca^2+^-ATPase dependent transporters and BKCa channels produces the vasodilation of blood vessels in the corpus cavernosum, leading to a penile erection [12]. cGMP binding to the allosteric sites of PDE-5 facilitates the binding of additional cGMP molecules to the active site of the enzyme and the consequent abolishment of cGMP activity (Figure 1) [9].

After an oral administration, sildenafil exerts its biological properties in few minutes, and its actions last around 12 h. The drug is metabolized by hepatic enzymes and possesses inhibitory properties toward CYP3A4, altering the metabolism of other classes of drugs such as antimycotic azoles and HIV protease inhibitors [13,14]. Common side effects are represented by rhinitis, headache, flushing, cardiovascular effects, and priapism. In addition, despite its selectivity towards the PDE-5 isozyme (IC50 = 3.5 nM), sildenafil possesses also the capability to bind PDE-6 (IC50 = 34 nM), an isoform specifically expressed in rod and cone cells of the retina determining visual side effects [15,16].

**Figure 2:**
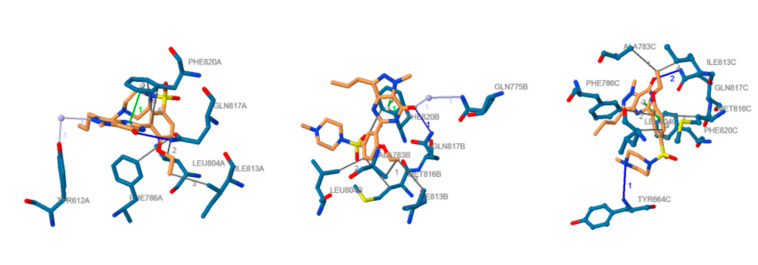
Docked position of sildenafil in the PDE-5 active site: ligand is represented in orange; amino acid residues in blue; hydrogen bonds are shown as solid blue lines; face-to-face stacking interaction in solid green lines; hydrogen bonds in dark solid grey lines; water bridges are represented in light solid grey lines. Image from the PLIP web service [20] using the PDB ID 2H42 [18].

Sildenafil is characterized by the presence of a pyrazo-lo[4,3]-d]pyrimidin-7(6H)-one nucleus that mimics the cGMP chemical structure. The pyrazole ring is decorated with alkyl substituents, whereas the pyrimidone ring is substituted with a phenyl ring bearing an ethoxy moiety and an N4-methylpiperazine-1-yl-sulfonyl moiety. Cocrystallization studies highlighted the binding mode of sildenafil to PDE-5 (Figure 2) [17]. The catalytic site of PDE-5 is characterized by the presence of four peculiar subsites. The M subsite (metal-binding subsite) possesses a zinc ion that takes interactions with histidine and aspartate amino acid residues and coordinates two water molecules. One aspartic residue and one water molecule coordinated by the zinc ion are also shared with a magnesium ion that takes interaction with four additional water molecules. The spatial disposition of the water molecules and amino acid residues involved in the interaction with zinc and magnesium ions retained an octahedral geometry [18]. The second water molecule coordinated by zinc and unbonded to magnesium is involved in a hydrogen bond with an additional water molecule whose spatial disposition is assured by hydrogen bonds with Tyr612 and the unsubstituted nitrogen atom of the pyrazole ring of sildenafil. This specific hydrogen bond network seems to play a pivotal role in the inhibition of the PDE-5; indeed, it is speculated that this water molecule acts as the nucleophile responsible for the hydrolysis of the phosphodiester bond of cGMP [19]. The Q pocket (core pocket) accommodates the heterocyclic ring of sildenafil. In this subsite, a Phe820 residue and the highly conserved Gln817 residue make a π-stacking interaction and a hydrogen bond with the amide function of the pyrimidinone ring, respectively. The hydrophobic subsite (H region) consists of a pocket in which highly lipophilic amino acid residues takes Van der Waals interactions with the ethoxyphenyl moiety linked to the heterocyclic core of sildenafil. Finally, a Tyr664 amino acid residue in the L region (lid pocket) undertakes a hydrogen bond with the N4 atom of the piperazine ring [18].

**Figure 3:**
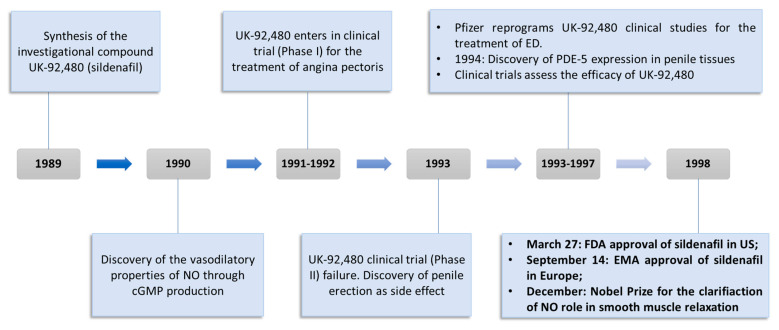
Timeline and milestones of sildenafil drug discovery.

The uncovering of sildenafil properties by Pfizer researchers represented one of the most resounding examples of serendipity in the drug discovery field (Figure 3) [11,21]. As a matter of fact, the cardiovascular research group operating in Pfizer in 1989 was looking for new drugs exploitable for the treatment of angina pectoris, a pathological condition caused by a temporary spasm of the coronary arteries with the consequent reduction of oxygen flow into the heart tissue [22]. The first clinical trials highlighted that UK-92,480 (sildenafil investigational code) did not possess any advantage when compared with other drugs commonly used for the treatment of angina pectoris, such as nitrates [23]. Indeed, doses of UK-92,480 administered intravenously or orally ranging from 20 to 200 mg weakly modified the hemodynamic parameters and potentiated the effects of nitrates. In response to these findings, UK-92,480 seemed to be not effective for the goal of the study, and Pfizer researchers started to fear that the drug development of UK-92,480 could suffer a setback. Unexpectedly, among the limited number of side effects detected during these studies, penile erection resulted as the most surprising [24]. At the time of the research, ED was considered as a condition primarily originated by psychological disturbs and treated with invasive injections of vasodilating substances in the penile tissues [11]. Moreover, PDE-5 was known to be principally localized in platelets and vascular smooth muscle cells, whereas its localization in the erectile tissues was never properly investigated. A subsequent study brought to light the presence of this specific enzymatic isoform in erectile tissues [25], allowing a better comprehension of the physiological processes that regulate penile erection [26]. In addition, this discovery confirmed that ED could be treated with orally administrable PDE-5 inhibitors because of the specific expression of this isozyme in erectile tissues, paving the way for the potential placing on the market of a class of compounds exploitable for an unmet clinical need. After 21 separate additional clinical trials carried out from 1993 to 1996 performed on a total number of about 3000 men aged 19 to 87 years old [11], the efficacy and patient’s compliance of UK-92,480, later named as sildenafil, was definitely confirmed. These results determined UK-92,480 approval by the FDA in March 1998 in the United States and by the EMA in September 1998 [27] under the trade name of Viagra. The placing on the market of this drug represented a global market breakthrough for the treatment of ED, with more than USD 400 million earned only in 1998 and more than USD 1 billion.

**Figure 4:**
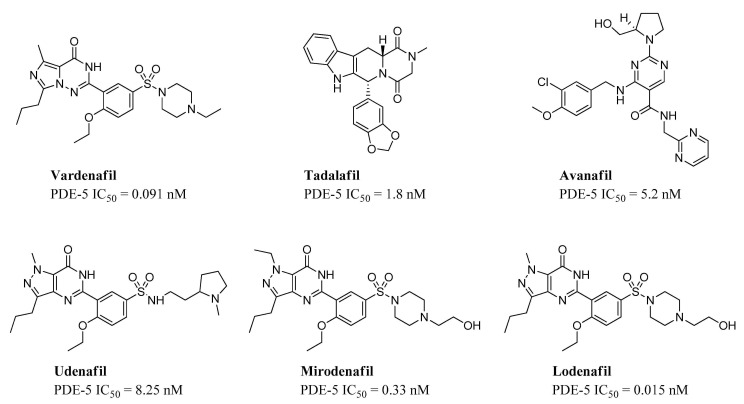
Chemical structures and IC_50_ values of commercial PDE-5 inhibitor.

In 2003, additional PDE-5 inhibitors entered the market (vardenafil and tadalafil), and in recent years, avanafil, mirodenafil, lodenafil, and udenafil have been approved in a limited number of countries (Figure 4) [28].

In 2010, sildenafil’s patent expired, and several industries started the production of this drug under generic names. Nevertheless, several clinical trials have been carried out in order to assess the efficacy of sildenafil for the treatment of other disabling pathologies [29,30,31,32].

## 2. Drug Repurposing Approach for the Identification of New Therapeutic Application

In spite of the increased understanding of prevention, diagnosis, therapy, and prognosis of human maladies, translation of this whole set of knowledge into new drugs has been far slower than estimated. A new drug discovery project generally starts with an unmet clinical need as the primary driving motivation. Initial efforts often occur in academia producing data to support a hypothesis that may result in the identification of a new target or a new therapeutic approach in a specific disease. In our time, however, drug discovery and development processes are resource- and time intensive and highly multifaceted requiring multidisciplinary profiles and innovative approaches. The attrition rate is another relevant aspect that the global pharmaceutical industry has to take into serious consideration when approaching a new discovery project. The latest estimations suggest that it takes more than 10 years and around USD 2 billion for a new drug to reach the market. There is growing pressure to set up cheaper and more effective ways to bring safe and efficacious drugs to the market. Within this framework, the drug discovery process is unceasingly experiencing changes and adjustments to achieve improvements in efficiency, productivity, and profitability. In this context, the so-called drug repositioning (or repurposing) process is attracting growing interest [33]. This strategy implies the identification of new therapeutic applications different from the original regulatory indication for approved or investigational drugs. The benefits of this strategy include tremendous savings of time and money, low risk of failure since the majority of preclinical and clinical trials, safety assessment, and, sometimes, pharmaceutical formulation have been completed. Finally, yet importantly, repurposed drugs may highlight novel targets and pathways that can be further investigated. In the past, the most significant examples of drug repurposing have been mainly based on serendipity rather than on a systematic approach. Once an off-target or a new on-target effect was detected, it was the object of further investigation and/or commercial exploitation. An outstanding example is represented by Zidovudine, which was originally developed as an anticancer agent but later became the first FDA-approved drug for the treatment of HIV after being identified from an in vitro screening of compound libraries [34]. Other remarkable examples include thalidomide, which was originally developed for morning sickness, and later, on the basis of pharmacological analysis, was approved for the treatment of erythema nodosum leprosum and multiple myeloma [35]. Minoxidil, originally indicated for the treatment of hypertension, was discovered by means of a retrospective clinical analysis. However, sildenafil represents maybe the foremost example. Originally investigated for angina, it represents maybe a perfect example of retrospective clinical analysis. Sildenafil was repurposed by Pfizer for the first time in the late 1990s for the management of ED. By 2014 it held the market-lead with a 47% share of the ED drug market and a worldwide sales calculation of around USD 2 billion [36]. Soon after its approval as Viagra, the discovery of the upregulation of PDE5 gene expression in pulmonary hypertensive lungs boosted further preclinical and clinical studies on sildenafil to test the role of PDE5 selective inhibitors in lung diseases [37]. Later, in 2005, the drug was repurposed once more for the treatment of pulmonary arterial hypertension and approved under the trade name Revatio [12,38]. Recently, other indications for which sildenafil has been studied include Raynaud’s disease, digital ulcer, heart failure, hypertensive cardiac hypertrophy, cerebral circulation, and different types of cancers including lung and colorectal malignancies [39,40].

## 3. In Vitro and In Vivo Applications of Sildenafil in Cancer Treatment

Many studies reported the use of sildenafil in combination with chemotherapeutic agents in the treatment of a variety of cancers (Table 1). Das et al. reported an increase in chemotherapeutic efficacy of DOX when coadministered with sildenafil in vitro on PC-3 and DU145 human prostate cancer cells. It was shown that combination therapy resulted in a relatively higher apoptotic rate on tumor cells by enhancing ROS generation, reducing B-cell lymphoma-extra large (Bcl-xL) expression, phosphorylating BAD, and upregulating caspase-3 and caspase-9 activities [41]. Further investigations on the molecular mechanisms involved in the sensitization of prostate cancer cells by sildenafil outlined the role of CD95 in DOX-mediated apoptosis [42]. The effect of sildenafil in enhancing the anticancer properties of DOX was eliminated when CD95 apoptosis-inducing death receptor was knocked down using siRNA. However, this was not the case when cells were treated with DOX alone. In addition, the combination therapy induced downregulation of Fas-associated phosphatase-1 (FAP-1) expression, a known inhibitor of CD95-mediated apoptosis, increasing cellular death and reducing tumor viability. Moreover, cells cotreated with sildenafil and DOX showed a reduced expression of both long and short forms of caspase-8 regulating enzymes Fas-associated death domain (FADD) interleukin-1-converting enzyme (FLICE)-like inhibitory protein (FLIP-L and -S), which are involved in the regulation of cellular apoptosis, compared to DOX-monotherapy [42]. Comparable results were reported for using the same therapeutic combination in the treatment of 4T1 murine breast cancer cells where synergistic activity was observed [43]. The outlined mechanisms clearly demonstrate the improved cytotoxic activity of DOX when combined with sildenafil, thereby potentially improving the clinical response and patient survival rate whilst ameliorating DOX toxic side effects. In vitro studies examining the potentiation of the antitumor activity of cisplatin when given in conjugation with sildenafil on MCF-7 human breast cancer cells showed a dose-dependent cytotoxic effect of sildenafil illustrating its potentiation effect on the chemotherapeutic agent [44]. Similar results were obtained upon cotreatment of MCF-7 and MDA-MB-468 human breast cancer cells with cisplatin and sildenafil, which was accompanied by a significant increase in accumulation of ROS into the extracellular environment in both breast adenocarcinomas cell lines [45].

The effect of coadministration of vincristine and sildenafil on PC-3 and DU145 human prostate cancer cell lines showed that a significant increase in vincristine-induced mitotic arrest and mitotic index [46]. The probability of cells being held in metaphase was dramatically increased in presence of sildenafil. This was particularly relevant in the tripolar spindle and multiple spindle poles. Nevertheless, a nonsignificant decrease in the level of cytokinesis was observed when cells responsive to vincristine were treated with sildenafil. Interestingly, the phosphorylation of Bcl-2 with caspase activation amplification including caspase-3, -8, and -9, and cleavage of poly [ADP-ribose] polymerase 1 (PARP-1), a caspase-3 substrate, was markedly increased when sildenafil was coadministered with vincristine; a similar feat previously demonstrated when sildenafil was combined with DOX on prostate cell lines incurring the coherence between the results reported between different studies. Additionally, sildenafil was shown to enhance vincristine-induced perturbation of microtubule–kinetochore interactions incurring higher apoptotic effects [46].

Roberts et al. reported that combination therapy of curcumin and sildenafil may induce gastrointestinal tumor cell death in HCT116, HT29, HuH7, HEP3B, and HEPG2 human gastrointestinal tumor cells through endoplasmic reticulum stress, reactive oxygen/nitrogen species, and increasing autophagosome and autolysosome levels prompting cancer cellular death [47]. Similar results were obtained when studying the effect of coadministration of curcumin and sildenafil on immunocompetent BALB/c mice implanted with CT26 murine colorectal cancer cells in which the use of sildenafil and curcumin as chemoadjuvants significantly enhanced the cytotoxic effect of 5-fluorouracil and anti-PD1 immunotherapy in vivo [48]. Such properties clearly express the ability of sildenafil to enhance cytotoxic properties of chemotherapeutic modalities as well as larger immunotherapeutic treating complexes.

The therapeutic efficacy of docetaxel and sildenafil in advanced prostate cancer was investigated by stimulating nitric oxide—cyclic guanosine-3′,5′-monophosphate (NO-cGMP) signaling. Human prostatic cancer (C4-2B) cells revealed overexpression of functional phosphodiesterase type 5 (PDE5) and its role with NO for aberrant cGMP accumulation. It was suggested that a subtherapeutic dose of docetaxel and a physiologically achievable sildenafil concentration could induce synergistic activity by increasing cGMP and blocking cells at G0/G1, inhibiting cell growth and inducing apoptosis. Similar results were observed in syngeneic cell lines and Pten cKO derived tumoroids where an increase in caspase-3 and PARP cleavage was detected [49]. The combination treatment demonstrated a significant decrease in tumoroid size and growth, with loss of integrity, apoptosis, condensed structure, and structural blebbing [50]. A demonstration between the 3D model translation, compared to the 2D line, further suggests an enhanced probability for in vivo studies and clinical application on patients.

The cytotoxicity of sildenafil/crizotinib loaded poly(ethylene glycol)-poly(DL-lactic acid) (PEG-PLA) polymeric micelles on MCF-7 human breast cancer cell lines was studied. Micelles with an average size between 93 and 127 nm and an encapsulation efficiency percentage (EE%) of both medications (>70%) were prepared using the solvent displacement method. In vitro cytotoxicity assays using crizotinib alone displayed 22% cellular viability, compared to 10% only upon coadministration of sildenafil, i.e., a 2.2-fold decrease in cell viability, after treatment for 48 hrs. This was attributed to previous reports on the wide inhibitory effect of sildenafil on several ATP-binding cassette (ABC) efflux transporters, henceforth overcoming cancer cell resistance and promoting their apoptosis [51]. Codelivery of these medications using nanoparticles further decreased the cell viability to 4%, illustrating the potential impact of formulation designs on enhancing the therapeutic outcomes of this regimen [52]. While these results suggest that the application of the dual-therapy in the nano form has shown a significant impact on the 2D tumor cells, issues regarding the formulation stability, pharmacokinetics, biodistribution, and in vitro 3D model and in vivo replication should be assessed before such formulations progress into the clinical phases.

In a different study, nanostructured lipid carrier (NLCs) coloaded with DOX and sildenafil citrate and tagged with arginyl-glycyl-aspartic acid (RGD) were prepared and their effect on human lung carcinoma A549 cells was studied [53]. The drug-loaded NLCs were prepared by homogenization method producing an optimum formula having an average size, polydispersity index, zeta potential, and EE% for DOX and sildenafil of 80.5 nm, 0.23, −18.5, 56.04 ± 1.25% and 81.62 ± 3.14%, respectively. The use of coloaded NLCs induced higher cytotoxicity and cancer cell apoptosis, compared to the free drug. It was suggested that this may be due to the enhanced cellular uptake and accumulation of drugs associated with integrin-mediated endocytosis and ABC transporter inhibition. Real-time PCR also revealed that sildenafil reduced the expression of ABCC1 and nuclear factor erythroid 2 related factor 2 (Nrf2) proteins, which incurred an increased intracellular concentration of anticancer drugs, as previously reported. It would be interesting to further explore the effect of the DOX/sildenafil-loaded nanoparticle formulation on the degree of ROS production, caspases activation, and proapoptotic protein expression [53,54].

In vivo studies using athymic male BALB/cAnNCr-nu/nu mice bearing prostatic cancer showed that the coadministration of sildenafil increased the efficacy of DOX while reducing DOX-associated cardiac dysfunction [41]. Immunohistochemistry demonstrated that the active form of caspase-3 was induced in tumors from sildenafil- and DOX-treated mice, compared with DOX-treated or nontreated control groups, henceforth explaining the relatively higher tumor volume reduction with the cotreatment. Furthermore, Doppler echocardiography showed a marked improvement in the left ventricular fractional shortening (LVFS) and left ventricular ejection fraction (LVEF) with sildenafil–DOX cotreatment rather than DOX alone. These results suggest a relatively lower systemic cytotoxicity associated with the cotreatment compared to monotherapy [41].

Treatment of female Balb/c mice inoculated with 4T1 murine mammary carcinoma cells with sildenafil/DOX combination therapy also demonstrated a significant reduction of tumor growth [43]. It was suggested that this effect is due to a higher migration of effective immune cells to the tumor site due to the vasodilatory effects of sildenafil, rather than an inherent cytotoxic effect of the drug. The results were in correlation with in vitro studies which demonstrated the lack of anticancer properties of sildenafil. Animals treated with DOX–sildenafil combination showed a 4.7 reduction in tumor size with a 2.7-fold increase in drug concentrations in comparison to DOX alone. Interestingly, when DOX was loaded into styrene-maleic acid (SMA) micelles and administered to the mice after sildenafil treatment, it showed a statistically insignificant increase in tumor accumulation, compared to SMA–DOX alone. This was not the case when dioctadecyl-3,3,3′,3′-tetramethylindocarbocyanine perchlorate (DiI) was loaded in SMA micelles and codelivered with sildenafil, where a statistically significant threefold increase was observed, compared to SMA–DiI alone. This difference could be associated with the variable particle size and physicochemical characteristics associated between both formulations. It was reported that SMA–DOX had a much smaller size (14.59 nm), compared to SMA–DiI (134.12 nm), hence would be able to accumulate to a larger extent at the tumor site, compared to the larger SMA–DiI without the need of sildenafil as a chemoadjuvant. These results raise the question of whether the magnitude of sildenafil efficacy as a chemoadjuvant could be affected by varying the particle size and characteristics of the nanoparticles used. A more holistic comparison would be to use comparable particles with close physicochemical properties to overcome such limitations [43].

Similarly, other in vivo studies using a combination of sildenafil and cisplatin showed a significant decrease in tumor volume in mice bearing breast cancer tumor, compared to the control group. Investigation of the local tissue microenvironment, apoptosis, and proliferation of the tumor cells after treatment with combination therapy showed an increase in caspase-3 levels with a considerable decrease in tumor necrosis factor-α contents, angiogenin, and vascular endothelial growth factor expression. However, the expression of Ki-67 nuclear protein which is usually present during the late G1, S, G2, and M phases of the cell cycle failed to show any significant changes when compared to the control group [44].

Muniyan et al. orthotopically implanted luciferase-labeled C4-2B cells into the dorsolateral lobe of the prostate in immunodeficient mice to investigate the therapeutic efficacy of coadministration of docetaxel and sildenafil in advanced prostate cancer [50]. The therapeutic combination significantly lowered tumor weight, compared to docetaxel alone. Further exploration in the molecular pathways responsible for this phenomenon identified a lower percentage of Ki67-positive nuclei and a higher frequency of cleaved caspase-3 positive cells, compared to groups treated with monotherapy, thus promoting apoptosis and tumor regression [50]. Likewise, Hsu et al. reported the synergistic effects between vincristine and sildenafil in PC-3-derived cancer xenografts in nude mice, demonstrating a decrease in tumor weight, compared to the single chemotherapeutic agent [46].

## 4. The Role of Sildenafil in Circumventing Anticancer Drug Resistance

MDR is a complex process in which cancer cells evolve to evade the deleterious effects of anticancer chemotherapy. A plethora of biological strategies had been described in association with the development of MDR. Enhanced drug metabolism, gene amplification, increase in DNA damage repair, epigenetic regulation of the drug targets, and autophagy all have been described.

Among different processes of drug resistance, overexpression of active transporters that actively efflux substrates of different chemical/biological natures is the most studied pathway, notably, the increase of drug efflux pumps ATP-binding cassette (ABC) transporters [55,56,57,58,59]. ABC transporter comprises ABCs (multidrug resistance-associated proteins (MRPs)), ABCB1 (P-glycoprotein/MDR1), and ABCG2 (BCRP/MXR/ABCP)) all were reported to be overexpressed in cancer developing the MDR. This superfamily transporter system mainly consists of integral membrane proteins. These proteins convert the energy that comes from ATP hydrolysis into the translocation of substrates across the membrane’s bilayer either into the cytoplasm or out of the cytoplasm. This movement is facilitated by a pair of transmembrane domains (TMDs), which when overexpressed in cancer cells, contribute to cell drug resistance by pumping out the intracellular drugs and therefore decreasing their cellular uptake and effect [60]. cGMP was implicated as a substrate for ATP-binding cassette (ABC) transporters in the multidrug resistance (MDR) cancer cells [58,59]. Accordingly, sildenafil was investigated as a potential player for reversing MDR in cancer cells.

Sildenafil increased the level of the second messenger’s cGMP through inhibition of PDE5, which is considered to be substrates for ABCC4/human MDR protein 4 (MRP4) and ABCC5/human MDR protein5 (MRP5), resulting in inhibition of the efflux pump activity. Furthermore, it inhibited the activity of ABC transporters such as ABCB1 and ABCG2, thereby increasing the sensitivity of MDR cells to various drugs. Moreover, the suppression of PDE5 could activate the cGMP-PKG pathway that mediates many processes causing cellular apoptosis or growth suppression (cell cycle arrest) of cancer cells [61].

Shi et al. demonstrated the effect of sildenafil on ABC transporters using ABC-mediated MDR on cancer cells. The cytotoxicity assays and drug accumulation results showed that sildenafil remarkably sensitized the ABCB1-overexpressing cells to the ABCB1 substrates (colchicine, vinblastine, and paclitaxel) with a high accumulation rate of the paclitaxel inside the cells. A similar effect on ABCG2-overexpressing cells was noted in relation to the substrates (flavopiridol, mitoxantrone, and SN-38) with a significant accumulation of mitoxantrone. In contrast, sildenafil had no effect on ABCC1-overexpressing cells and its tested substrate (vincristine). Altogether, these data strongly suggest a potential role for sildenafil in reversing anticancer drug resistance [62].

## 5. Sildenafil and Anticancer Drug Delivery through EPR Augmentation

PDE5 inhibitors such as sildenafil had demonstrated their effect on smooth muscle layers of blood vessels leading to vasodilation in tissues that express the specific isoenzyme. Indeed, one known side effect of this class of drugs is systemic hypotension that denotes the susceptibility of normal vascular cell types to PDE5 inhibitors [63].

Smooth muscle relaxation thereby modulates vascular endothelial permeability that increases the inflow of blood to the normal and pathological tissues such as inflamed tissues and tumor tissues, leading to the accumulation of nanoparticles of molecular weight exceeding 40 kD and augmenting preferential drug targeting in the diseased tissues such as tumors. This accumulation normally occurs due to the abnormalities in tumor vascularity due to poorly aligned and faulty vascular endothelial cells that have wide fenestrations of up to 4 μm [64,65,66]. Traditionally, the EPR effect involves two aspects. First, the drug preferential biodistribution is related to the size of the drug and the delivery vehicle applied to achieve the differential accumulation of the drug in tumor tissues. As the size of the drug and the delivery vehicle is more than the limit of the renal excretion threshold, nanoparticles usually exhibit increased plasma half-life. Second, the EPR effect involves retention of the nano-based system due to the lack of efficient lymphatic clearance [67,68,69].

Unfortunately, a very slim volume of existing literature examines the response of tumor vasculature to PDE5 inhibitors. PDE inhibition could potentially result in improvement of blood supply to the tumor tissues through similar mechanisms employed for ED.

In order to augment the EPR effect of macromolecular drugs, sildenafil needs to be preferentially applied locally to the tumor site. Relevant work had been pioneered by Maeda et al., in which they applied the nitric oxide donor Lipiodol^®^ through the arterial catheter to the tumor feeding artery with reported success in the management of clinically advanced cases of primary and secondary liver tumors [70]. This early experience proved that in order to selectively utilize a vasodilating agent to improve the EPR effect, the vasodilation needs to be restricted to the blood supply in the close vicinity of the tumor tissues, otherwise widespread vasodilation can enhance the delivery of the nanoconstructs to other off-target tissues and induce systemic hypotension.

Greish et al. demonstrated that using sildenafil in conjunction with DOX increased the concentration of the anticancer drug in tumor tissues by 2.7 folds, and eventually resulted in 4.7 folds improved anticancer activity against the 4T1 breast cancer in mice. This work suggests a positive effect of PDE5 inhibitors to further augment enhanced permeability and retention (EPR) effect on EPR effect [43]. A relevant study by Black et al. demonstrated the effect of PDE5 inhibitors on enhancing tumor vascular permeability in the brain tumor model of 9L gliosarcoma-bearing in rats. Sildenafil administration increased the tumor capillary permeability in comparison to the normal brain capillaries, which showed no significant increase in vascular permeability. Additionally, the study proved a synergistic effect of the use of anthracycline chemotherapy combined with the sildenafil and further improved the survival by nearly twofold longer than the group treated with the chemotherapeutic agent alone [71]. Another work by Zhang et al. provided further direct evidence of the potential of PDE 5 inhibitors in augmenting EPR-mediated anticancer chemotherapy in vivo. In their study, the team employed a combined micelle incorporating both cisplatin and sildenafil. The team proposed that tumor acidity can preferentially release the PDE5 inhibitor from the micelle, further augmenting its concentration in tumor tissues. This strategy was proved effective in increasing both drug accumulation and anticancer activity in the tested cancer model of B16F10 melanoma in C57BL/6 mice, altogether indicating a potential and promising rule for PDE5 inhibitors in augmenting EPR-based anticancer drug delivery [72].

It is noteworthy to mention that sildenafil application for augmenting local tumor tissue concentrations of chemotherapy is not exclusive to nanosized molecules. It can similarly increase the local concentration of conventional chemotherapeutic agents [43]. However, small molecules traverse barriers freely into the tumor or the normal tissue and immediately disappear from the tumor or the normal tissue by diffusion primarily into blood capillaries. Therefore, the residence time of conventional small molecular drugs in cancer tissue is usually counted in minutes, while that of nanosized molecules by days to weeks, owing to the retention aspect in the EPR effect. Accordingly, since tumor tissues lack functional lymphatics, the enhanced delivery of bioactive nanosize molecules in the tumor is usually retained for considerable durations.

## 6. Clinical Studies

The use of sildenafil in the management of different types of cancer has been the subject of various clinical trials (http://www.clinicaltrials.gov accessed on 1 March 2021) (Table 2). A number of clinical trials such as NCT00142506, NCT00544076, NCT00057759, and NCT00511498, evaluated the use of sildenafil alone or in combination with alprostadil or hyperbaric oxygen therapy in the management of ED. Those trials focused on restoring the erectile function for patients with prostate cancer after radiotherapy or nerve-sparing prostatectomy. Clinical trial NCT02106871 was designed to assess the use of sildenafil monotherapy in the treatment of fatigue in patients with pancreatic cancer. It is suggested that sildenafil increases protein synthesis, alters protein expression and nitrosylation, and reduces fatigue in human skeletal muscle especially in patients with reduced skeletal muscle functions [73]. The concept has yet to be clinically tested as the study was terminated due to a lack of funds. The ability of sildenafil monotherapy to improve renal functions in patients with kidney cancer after partial nephrectomy and protect the kidney from the side effects of surgery was investigated in clinical trial NCT01950923. The study involved the oral administration of sildenafil to 30 patients prior to surgery, followed by assessment of kidney functions. The trial was completed but the results have yet to be reported. In clinical trial NCT00165295, sildenafil was tested in the treatment of Waldenstrom′s Macroglobulinemia (WM), a rare and incurable type of non-Hodgkin lymphoma. It was suggested that sildenafil blocks the function of several proteins necessary to the survival of cancer cells, and laboratory tests have shown that it can destroy WM cells [74]. The study involved 30 patients who received incremental doses of sildenafil orally for 2 years. The clinical trial has been completed with no reported side effects, but the complete results of the study are not published yet. Sildenafil was also tested for the treatment of Lymphangioma in pediatric patients in clinical trial NCT01290484. The results showed a significant decrease of lymphatic malformation in four out of seven patients included in the study after oral administration of sildenafil for 20 weeks with no observed complications in any subject [75].

The use of sildenafil as a chemoadjuvant in the treatment of different types of cancers was investigated. The clinical trial NCT01375699 investigated the use of sildenafil as a cardioprotective agent in female patients primarily with breast cancer treated with DOX against the cardiotoxic effects of the drug. Patients were given oral sildenafil daily for one week prior to the scheduled first dose of DOX. The treatment continued until 2 weeks after the last scheduled dose of DOX and multiple biomarkers for cardiotoxicity were measured [76]. The results showed that adding sildenafil to DOX chemotherapy is safe and well tolerated but did not significantly improve cardiac protection during chemotherapy when compared to the control group. The trial NCT00752115 used sildenafil combination with chemotherapeutic agents such as carboplatin and paclitaxel in patients with advanced non-small-cell lung cancer to improve the biodistribution and efficacy of the chemotherapeutic agents. Patients received a weekly dose of 50 mg sildenafil orally and progression-free survival was monitored. The phase I clinical trial NCT02466802 assessed the use of regorafenib in combination with sildenafil in patients with progressive advanced solid tumors. The study results showed that the drug combination is safe and that the lethality of this combination could be enhanced in vitro and in vivo by the addition of neratinib to the treatment regimen in a colorectal cancer model. Accordingly, it was further recommended to perform a phase I trial in colorectal cancer patients using the combination of the three drugs [77]. The phase II clinical study NCT01817751 is currently investigating the use of sildenafil as a chemoadjuvant in the treatment of patients with recurrent high-grade glioma. Orally administered sildenafil twice a day for four weeks is used in combination with sorafenib and valproic acid to test its ability to increase the concentration of the chemotherapeutic agents in the brain and preventing the growth of tumor cells by blocking BCG2 drug efflux pump in the blood–brain barrier.

It is very clear that most of the clinical trials focused on using sildenafil as a chemoadjuvant to reverse side effects associated with chemotherapy such as ED or cardiotoxicity. This means that much of the potential for the use of sildenafil in the treatment of different types of cancer remains theoretical, lacking solid clinical evidence. More clinical trials are still required to test the possibility to use sildenafil in circumventing anticancer drug resistance and as an EPR augmentation tool for enhancement of anticancer drug delivery.

## 7. Conclusions and Future Recommendations

The paradigm of drug repurposing remains of significant interest for the pharmaceutical and health care communities. A deeper understanding of the molecular pathology and pharmacology of the current therapeutic entities in the market plays an important role in the utilization of current resources in the management of various diseases. Further to Meade’s visionary discovery of EPR, he recommended further augmentation of this key biological effect by manipulating vascular dynamics at macro- and micro-organizational levels. Sildenafil has demonstrated its ability in enhancing anticancer drug delivery through the EPR effect, prompting significant elevation of intratumoral drug concentrations and subsequent cellular death. In addition, sildenafil has demonstrated its implication in the modulation and potentiation of chemotherapeutic agents in a range of different types of cancer. This has been outlined in several in vitro and in vivo studies through the downregulation of Bcl-xL and FAP-1 expression, enhancing ROS generation, phosphorylating BAD and Bcl-2, upregulating caspase-3,8,9 activities, blocking cells at G0/G1 cell cycle phase, overcoming cancer cell resistance by inhibiting several ABC transporters through cGMP elevation, and increasing autophagosome and autolysosome levels; inducing tumor cell death.

Despite several clinical studies being underway, the need for further trials on patients remains of paramount importance to further understand the clinical impact they may perceive. These studies could possibly include the application of novel drug delivery formulations for combination therapies such as passively and actively targeting nanoparticles, external stimuli-responsive systems using light, focused ultrasound, and magnetic fields to release the drug therapy at the desired site of action, and controlled-release formulations where sildenafil may precede the chemotherapeutic agent, inducing its chemosensitizing action first and promoting higher cytotoxicity action of the latter. Such systems could certainly increase the efficacy and safety profiles of current oncological agents, enhancing the patient’s quality of life and achieving a definite therapeutic outcome.

## Figures and Tables

**Table 1:** Examples of in vitro and in vivo studies for the effect of sildenafil in different types of cancer.

Cancer	Type of Study	Tumor Model	Therapy	Therapeutic Outcome	Ref.
Prostate Cancer	In vitro	PC-3 and DU145 prostate cancer cells	Sildenafil (10 μM)	No significant changes in % Cell death compared to control	[41]
			DOX (1.5 μM with PC-3 and 0.5 μM with DU145)	7.52% and 45.01% cell death in PC-3 and DU145 cells, respectively.	
			DOX (1.5 μM with PC-3 and 0.5 μM with DU145) + Sildenafil (10 μM)	18.71% and 56.82% cell death in PC-3 and DU145 cells, respectively.	
	In vivo	Athymic male BALB/cAnNCr-nu/nu mice injected with PC-3 cells and 50-μL matrigel matrices	DOX (1.5 mg/kg)	Tumor weight/Body weight ratio = 0.015	
			Intraperitoneal DOX (1.5 mg/kg) + Sildenafil (5 mg/kg) OR intraperitoneal DOX (3 mg/kg) + oral Sildenafil (10 mg/kg)	Tumor weight/Body weight ratio = 0.010	
Breast Cancer	In vitro	4T1 mammary carcinoma cells	DOX (1μM)	50% cell death	[43]
			Sildenafil (10,30,100μM)	No significant changes compared to control	
			DOX (1μM) + Sildenafil (1μM)	72.2% cell death	
			DOX (1μM) + Sildenafil (30μM)	91.9% cell death	
			DOX (1μM) + Sildenafil (100μM)	97.6% cell death	
	In vivo	Female Balb/c mice injected with 4T1 mammary carcinoma cells	DOX (5 mg/kg)	Tumor volume = 570%	
			Sildenafil (1 mg/kg)	Tumor volume = 400%	
			DOX (5 mg/kg) + Sildenafil (1 mg/kg)	Tumor volume = 121.3%	
Breast Cancer	In vitro	MCF-7 breast cancer cells	Sildenafil	IC_50_ = 14 µg/mL	[44]
			Cisplatin	IC_50_ = 4.43 µg/mL	
			Sildenafil + Cisplatin	IC_50_ = 3.98 µg/mL	
	In vivo	Swiss albino female mice injected with Ehrlich ascites carcinoma (EAC) cells	Sildenafil (5 mg/kg)	30.4% decrease in tumor volume	
			Cisplatin (7.5 mg/kg)	58.8% decrease in tumor volume	
			Sildenafil (5 mg/kg) + Cisplatin (7.5 mg/kg)	79% decrease in tumor volume	
Colorectal Cancer	In vitro	HT-29, SW480, SW620, HCT116 and SW1116 colorectal cancer cells	Sildenafil	IC_50_ (72hrs) = 190.91 μM in HT-29 217.27 μM SW480 206.68 μM SW620 246.20 μM HCT116 271.22 μM SW1116	[39]
	In vivo	Balb/c nude mice injected with SW480 or HCT116 colorectal cancer cells	Sildenafil (50 mg/kg) and (150 mg/kg)	In SW480, 40.1% and 57.8% tumor inhibition with 50 mg/kg and 150 mg/kg, respectively.	
				In HCT116, 13.3% and 61.4% tumor inhibition with 50 mg/kg and 150 mg/kg, respectively.	
Prostate Cancer	In vivo	Nude mice were injected with PC-3 prostate cancer cells	Sildenafil (10 mg/kg)	Tumor weight = 969.9 ± 92.2 mg	[46]
			Vincristine (0.5 mg/kg)	Tumor weight = 623.5 ± 132.2 mg	
			Sildenafil (10 mg/kg) + Vincristine (0.5 mg/kg)	Tumor weight = 207.6 ± 36.7 mg	
Breast Cancer	In vitro	MCF-7 Breast cancer cells	Sildenafil	No significant changes in % cell viability compared to control	[52]
			Crizotinib	IC_50_ = 34.19 and 22% cell viability	
			Crizotinib + Sildenafil	IC_50_ = 3.34 and 10% cell viability	
			Blank PEG-PLA micelles	No significant changes in % cell viability compared to control	
			Crizotinib loaded PEG-PLA micelles	14% cell viability	
			Crizotinib (55.25 μM)/Sildenafil (40.33 μM)- coloaded PEG-PLA micelles	4% cell viability	
Lung Cancer	In vitro	A549 human lung carcinoma cells	DOX	29.87% cell death	[53]
			DOX + Sildenafil	34.69% cell death	
			DOX/Sildenafil-coloaded NLC	38.37% cell death	
			DOX/Sildenafil-coloaded NLC-RGD	44.32% cell death	

**Table 2:** Examples of clinical trials using sildenafil in treatment of different types of cancers *.

Types of Cancer	Treatment	Objective	Stage
Pancreatic Cancer	Sildenafil	Management of fatigue in cancer patient undergoing chemotherapy	Phase I
Non-small Cell Lung Cancer	Sildenafil, Paclitaxel, Carboplatin	Improvement in distribution and efficacy of cytotoxic anticancer agents	Phase II, III
Prostate Cancer	Sildenafil	Management of ED during and after radiotherapy with or without hormone Therapy	Phase III
	Sildenafil, Alprostadil	Management of ED post-operatively in patients undergoing nerve-sparing robotic-assisted radical prostatectomy	Phase III
	Sildenafil	Investigate the effect of dosage regimen on ED in patients after nerve-sparing laparoscopic radical prostatectomy	Not applicable
	Sildenafil, Hyperbaric oxygen therapy	Management of ED in patients after nerve-sparing radical retropubic prostatectomy	Phase IV
Solid Tumor	Regorafenib Sildenafil	Investigation of the antitumor effects of the regorafenib and sildenafil combination, the pre-treatment expression of phosphodiesterase type 5 (PDE5) in tumor samples, and the impact of sildenafil on the pharmacokinetics of regorafenib	Phase I
Kidney Tumor	Sildenafil	Improving Postoperative Kidney Function in Patients With Kidney Cancer undergoing Robotic Partial Nephrectomy	Phase I
Colorectal Cancer	Sildenafil Vacuum erection device (VED)	Management of ED After Laparoscopic Resection	Phase IV
Breast Cancer	Sildenafil Doxorubicin	Improving antitumor effects of DOX and protection from cardiac toxicity	Phase I
Brain Cancer and Glioblastoma	Sildenafil Sorafenib Tosylate Valproic Acid	Increase the concentration of anticancer drug in the brain and stop the growth of tumor cells by blocking BCG2 drug efflux pump in the blood–brain barrier	Phase II
Waldenstrom Macroglobulinemia	Sildenafil	Treatment by blocking the function of several proteins necessary to the survival of cancer cells	Phase II
Myelodysplastic Syndrome (MDS)	Nivolumab Cytarabine Sildenafil	Studying the pathogenesis and resistance of myelodysplastic syndrome using combination therapy	Phase I, II

* Source: https://clinicaltrials.gov/ (accessed on 1 March 2021).

## References

[1] Utiger R.D. (1998). A Pill for Impotence. N. Engl. J. Med..

[2] Terrett N.K., Bell A.S., Brown D., Ellis P. (1996). Sildenafil (Viagra(TM)), a potent and selective inhibitor of type 5 CGMP phosphodiesterase with utility for the treatment of male erectile dysfunction. Bioorg. Med. Chem. Lett..

[3] Francis S.H., Blount M.A., Corbin J.D. (2011). Mammalian cyclic nucleotide phosphodiesterases: Molecular mechanisms and physiological functions. Physiol. Rev..

[4] Rotella D.P. (2002). Phosphodiesterase 5 inhibitors: Current status and potential applications. Nat. Rev. Drug Discov..

[5] Beavo J.A. (1995). Cyclic nucleotide phosphodiesterases: Functional implications of multiple isoforms. Physiol. Rev..

[6] Andersson K.E. (2018). PDE5 inhibitors—Pharmacology and clinical applications 20 years after sildenafil discovery. Br. J. Pharmacol..

[7] Blount M.A., Beasley A., Zoraghi R., Sekhar K.R., Bessay E.P., Francis S.H., Corbin J.D. (2004). Binding of tritiated sildenafil, tadalafil, or vardenafil to the phosphodiesterase-5 catalytic site displays potency, specificity, heterogeneity, and cGMP stimulation. Mol. Pharmacol..

[8] Corbin J.D., Francis S.H. (1999). Cyclic GMP phosphodiesterase-5: Target of sildenafil. J. Biol. Chem..

[9] Corbin J.D., Turko I.V., Beasley A., Francis S.H. (2000). Phosphorylation of phosphodiesterase-5 by cyclic nucleotide-dependent protein kinase alters its catalytic and allosteric cGMP-binding activities. Eur. J. Biochem..

[10] Essayan D.M. (2001). Cyclic nucleotide phosphodiesterases. J. Allergy Clin. Immunol..

[11] Goldstein I., Burnett A.L., Rosen R.C., Park P.W., Stecher V.J. (2019). The Serendipitous Story of Sildenafil: An Unexpected Oral Therapy for Erectile Dysfunction. Sex. Med. Rev..

[12] Ghofrani H.A., Osterloh I.H., Grimminger F. (2006). Sildenafil: From angina to erectile dysfunction to pulmonary hypertension and beyond. Nat. Rev. Drug Discov..

[13] Carson C.C., Lue T.F. (2005). Phosphodiesterase type 5 inhibitors for erectile dysfunction. BJU Int..

[14] Barbaro G., Scozzafava A., Mastrolorenzo A., Supuran C. (2005). Highly Active Antiretroviral Therapy: Current State of the Art, New Agents and Their Pharmacological Interactions Useful for Improving Therapeutic Outcome. Curr. Pharm. Des..

[15] Yafi F.A., Sharlip I.D., Becher E.F. (2018). Update on the Safety of Phosphodiesterase Type 5 Inhibitors for the Treatment of Erectile Dysfunction. Sex. Med. Rev..

[16] Laties A.M., Fraunfelder F.T., Flach A.J., Tasman W. (1999). Ocular safety of Viagra, (sildenafil citrate). Trans. Am. Ophthalmol. Soc..

[17] Wang H., Liu Y., Huai Q., Cai J., Zoraghi R., Francis S.H., Corbin J.D., Robinson H., Xin Z., Lin G. (2006). Multiple conformations of phosphodiesterase-5: Implications for enzyme function and drug development. J. Biol. Chem..

[18] Sung B.J., Hwang K.Y., Jeon Y.H., Lee J.I., Heo Y.S., Kim J.H., Moon J., Yoon J.M., Hyun Y.L., Kim E. (2003). Structure of the catalytic domain of human phosphodiesterase 5 with bound drug molecules. Nature.

[19] Supuran C., Mastrolorenzo A., Barbaro G., Scozzafava A. (2006). Phosphodiesterase 5 Inhibitors—Drug Design and Differentiation Based on Selectivity, Pharmacokinetic and Efficacy Profiles. Curr. Pharm. Des..

[20] Salentin S., Schreiber S., Haupt V.J., Adasme M.F., Schroeder M. (2015). PLIP: Fully automated protein-ligand interaction profiler. Nucleic Acids Res..

[21] Ban T.A. (2006). The role of serendipity in drug discovery. Dialogues Clin. Neurosci..

[22] Deckers J.W. (2013). Classification of myocardial infarction and unstable angina: A re-assessment. Int. J. Cardiol..

[23] Jackson G., Benjamin N., Jackson N., Allen M.J. (1999). Effects of sildenafil citrate on human hemodynamics. Am. J. Cardiol..

[24] Boolell M., Allen M.J., Ballard S.A., Gepi-Attee S., Muirhead G.J., Naylor A.M., Osterloh I.H., Gingell C. (1996). Sildenafil: An orally active type 5 cyclic GMP-specific phosphodiesterase inhibitor for the treatment of penile erectile dysfunction. Int. J. Impot. Res..

[25] Wallis R.M., Corbin J.D., Francis S.H., Ellis P. (1999). Tissue distribution of phosphodiesterase families and the effects of sildenafil on tissue cyclic nucleotides, platelet function, and the contractile responses of trabeculae carneae and aortic rings in vitro. Am. J. Cardiol..

[26] Ignarro L.J., Bush P.A., Buga G.M., Wood K.S., Fukuto J.M., Rajfer J. (1990). Nitric oxide and cyclic GMP formation upon electrical field stimulation cause relaxation of corpus cavernosum smooth muscle. Biochem. Biophys. Res. Commun..

[27] Perry M.J., Higgs G.A. (1998). Chemotherapeutic potential of phosphodiesterase inhibitors. Curr. Opin. Chem. Biol..

[28] Hatzimouratidis K., Salonia A., Adaikan G., Buvat J., Carrier S., El-Meliegy A., McCullough A., Torres L.O., Khera M. (2016). Pharmacotherapy for Erectile Dysfunction: Recommendations From the Fourth International Consultation for Sexual Medicine (ICSM 2015). J. Sex. Med..

[29] Bermejo J., Yotti R., García-Orta R., Sánchez-Fernández P.L., Castaño M., Segovia-Cubero J., Escribano-Subías P., San Román J.A., Borrás X., Alonso-Gómez A. (2018). Sildenafil for improving outcomes in patients with corrected valvular heart disease and persistent pulmonary hypertension: A multicenter, double-blind, randomized clinical trial. Eur. Heart J..

[30] Galiè N., Ghofrani H.A., Torbicki A., Barst R.J., Rubin L.J., Badesch D., Fleming T., Parpia T., Burgess G., Branzi A. (2005). Sildenafil Citrate Therapy for Pulmonary Arterial Hypertension. N. Engl. J. Med..

[31] Kolb M., Raghu G., Wells A.U., Behr J., Richeldi L., Schinzel B., Quaresma M., Stowasser S., Martinez F.J. (2018). Nintedanib plus Sildenafil in Patients with Idiopathic Pulmonary Fibrosis. N. Engl. J. Med..

[32] Sastry B.K.S., Narasimhan C., Reddy N.K., Raju B.S. (2004). Clinical efficacy of sildenafil in primary pulmonary hypertension: A randomized, placebo-controlled, double-blind, crossover study. J. Am. Coll. Cardiol..

[33] Pushpakom S., Iorio F., Eyers P.A., Escott K.J., Hopper S., Wells A., Doig A., Guilliams T., Latimer J., McNamee C. (2018). Drug repurposing: Progress, challenges and recommendations. Nat. Rev. Drug Discov..

[34] Marwick C. (1987). AZT (zidovudine) just a step away from FDA approval for AIDS therapy. JAMA.

[35] Stewart A.K. (2014). How thalidomide works against cancer. Science.

[36] Rezaee M.E., Ward C.E., Brandes E.R., Munarriz R.M., Gross M.S. (2020). A Review of Economic Evaluations of Erectile Dysfunction Therapies. Sex. Med. Rev..

[37] Sanchez L.S., De La Monte S.M., Filippov G., Jones R.C., Zapol W.M., Bloch K.D. (1998). Cyclic-GMP-binding, cyclic-GMP-specific phosphodiesterase (PDE5) gene expression is regulated during rat pulmonary development. Pediatr. Res..

[38] Bhogal S., Khraisha O., Al Madani M., Treece J., Baumrucker S.J., Paul T.K. (2019). Sildenafil for Pulmonary Arterial Hypertension. Am. J. Ther..

[39] Mei X.L., Yang Y., Zhang Y.J., Li Y., Zhao J.M., Qiu J.G., Zhang W.J., Jiang Q.W., Xue Y.Q., Zheng D.W. (2015). Sildenafil inhibits the growth of human colorectal cancer in vitro and in vivo. Am. J. Cancer Res..

[40] Keats T., Rosengren R.J., Ashton J.C. (2018). The Rationale for Repurposing Sildenafil for Lung Cancer Treatment. Anticancer Agents Med. Chem..

[41] Das A., Durrant D., Mitchell C., Mayton E., Hoke N.N., Salloum F.N., Park M.A., Qureshi I., Lee R., Dent P. (2010). Sildenafil increases chemotherapeutic efficacy of doxorubicin in prostate cancer and ameliorates cardiac dysfunction. Proc. Natl. Acad. Sci. USA.

[42] Das A., Durrant D., Mitchell C., Dent P., Batra S.K., Kukreja R.C. (2016). Sildenafil (Viagra) sensitizes prostate cancer cells to doxorubicin-mediated apoptosis through CD95. Oncotarget.

[43] Greish K., Fateel M., Abdelghany S., Rachel N., Alimoradi H., Bakhiet M., Alsaie A. (2018). Sildenafil citrate improves the delivery and anticancer activity of doxorubicin formulations in a mouse model of breast cancer. J. Drug Target..

[44] El-Naa M.M., Othman M., Younes S. (2016). Sildenafil potentiates the antitumor activity of cisplatin by induction of apoptosis and inhibition of proliferation and angiogenesis. Drug Des. Dev. Ther..

[45] Hassanvand F., Mohammadi T., Ayoubzadeh N., Tavakoli A., Hassanzadeh N., Sanikhani N.S., Azimi A.I., Mirzaei H.R., Khodamoradi M., Goudarzi K.A. (2020). Sildenafil enhances cisplatin-induced apoptosis in human breast adenocarcinoma cells. J. Cancer Res. Ther..

[46] Hsu J.-L., Leu W.-J., Hsu L.-C., Ho C.-H., Liu S.-P., Guh J.-H. (2020). Phosphodiesterase Type 5 Inhibitors Synergize Vincristine in Killing Castration-Resistant Prostate Cancer Through Amplifying Mitotic Arrest Signaling. Front. Oncol..

[47] Roberts J.L., Poklepovic A., Booth L. (2017). Curcumin interacts with sildenafil to kill GI tumor cells via endoplasmic reticulum stress and reactive oxygen/nitrogen species. Oncotarget.

[48] Dent P., Booth L., Roberts J.L., Poklepovic A., Hancock J.F. (2020). (Curcumin + sildenafil) enhances the efficacy of 5FU and anti-PD1 therapies in vivo. J. Cell. Physiol..

[49] Morales J.C., Li L., Fattah F.J., Dong Y., Bey E.A., Patel M., Gao J., Boothman D.A. (2014). Review of poly (ADP-ribose) polymerase (PARP) mechanisms of action and rationale for targeting in cancer and other diseases. Crit. Rev. Eukaryot. Gene Expr..

[50] Muniyan S., Rachagani S., Parte S., Halder S., Seshacharyulu P., Kshirsagar P., Siddiqui J.A., Vengoji R., Rauth S., Islam R. (2020). Sildenafil potentiates the therapeutic efficacy of docetaxel in advanced prostate cancer by stimulating NO-cGMP signaling. Clin. Cancer Res..

[51] Chen J.J., Sun Y.L., Tiwari A.K., Xiao Z.J., Sodani K., Yang D.H., Vispute S.G., Jiang W.Q., Chen S.D., Chen Z.S. (2012). PDE5 inhibitors, sildenafil and vardenafil, reverse multidrug resistance by inhibiting the efflux function of multidrug resistance protein 7 (ATP-binding Cassette C10) transporter. Cancer Sci..

[52] Marques J.G., Gaspar V.M., Markl D., Costa E.C., Gallardo E., Correia I.J. (2014). Co-delivery of Sildenafil (Viagra(®)) and Crizotinib for synergistic and improved anti-tumoral therapy. Pharm. Res..

[53] Hajipour H., Ghorbani M., Kahroba H., Mahmoodzadeh F., Emameh R.Z., Taheri R.A. (2019). Arginyl-glycyl-aspartic acid (RGD) containing nanostructured lipid carrier co-loaded with doxorubicin and sildenafil citrate enhanced anti-cancer effects and overcomes drug resistance. Process. Biochem..

[54] Ji L., Li H., Gao P., Shang G., Zhang D.D., Zhang N., Jiang T. (2013). Nrf2 Pathway Regulates Multidrug-Resistance-Associated Protein 1 in Small Cell Lung Cancer. PLoS ONE.

[55] Mansoori B., Mohammadi A., Davudian S., Shirjang S., Baradaran B. (2017). The Different Mechanisms of Cancer Drug Resistance: A Brief Review. Adv. Pharm. Bull..

[56] Di Nicolantonio F., Mercer S.J., Knight L.A., Gabriel F.G., Whitehouse P.A., Sharma S., Fernando A., Glaysher S., Di Palma S., Johnson P. (2005). Cancer cell adaptation to chemotherapy. BMC Cancer.

[57] Vasiliou V., Vasiliou K., Nebert D.W. (2009). Human ATP-binding cassette (ABC) transporter family. Hum. Genom..

[58] Cree I.A., Knight L., di Nicolantonio F., Sharma S., Gulliford T. (2002). Chemosensitization of solid tumor cells by alteration of their susceptibility to apoptosis. Curr. Opin. Investig. Drugs.

[59] Tewari K.S., Eskander R.N., Monk B.J. (2015). Development of Olaparib for BRCA-Deficient Recurrent Epithelial Ovarian Cancer. Clin. Cancer Res. Off. J. Am. Assoc. Cancer Res..

[60] Gottesman M.M., Ambudkar S. (2001). V Overview: ABC transporters and human disease. J. Bioenerg. Biomembr..

[61] Shi Z., Tiwari A.K., Shukla S., Robey R.W., Singh S., Kim I.-W., Bates S.E., Peng X., Abraham I., Ambudkar S.V. (2011). Sildenafil reverses ABCB1- and ABCG2-mediated chemotherapeutic drug resistance. Cancer Res..

[62] Shi Z., Tiwari A.K., Patel A.S., Fu L.W., Chen Z.S. (2011). Roles of sildenafil in enhancing drug sensitivity in cancer. Cancer Res..

[63] Preston I.R., Klinger J.R., Houtches J., Nelson D., Farber H.W., Hill N.S. (2005). Acute and chronic effects of sildenafil in patients with pulmonary arterial hypertension. Respir. Med..

[64] Yuan F., Salehi H.A., Boucher Y., Vasthare U.S., Tuma R.F., Jain R.K. (1994). Vascular permeability and microcirculation of gliomas and mammary carcinomas transplanted in rat and mouse cranial windows. Cancer Res..

[65] Folkman J. (1995). Angiogenesis in cancer, vascular, rheumatoid and other disease. Nat. Med..

[66] Hashizume H., Baluk P., Morikawa S., McLean J.W., Thurston G., Roberge S., Jain R.K., McDonald D.M. (2000). Openings between defective endothelial cells explain tumor vessel leakiness. Am. J. Pathol..

[67] Matsumura Y., Maeda H. (1986). A new concept for macromolecular therapeutics in cancer chemotherapy: Mechanism of tumoritropic accumulation of proteins and the antitumor agent smancs. Cancer Res..

[68] Seymour L.W., Miyamoto Y., Maeda H., Brereton M., Strohalm J., Ulbrich K., Duncan R. (1995). Influence of molecular weight on passive tumour accumulation of a soluble macromolecular drug carrier. Eur. J. Cancer.

[69] Greish K., Sawa T., Fang J., Akaike T., Maeda H. (2004). SMA-doxorubicin, a new polymeric micellar drug for effective targeting to solid tumours. J. Control. Release.

[70] Nagamitsu A., Inuzuka T., Greish K., Maeda H. (2007). SMANCS dynamic therapy for various advanced solid tumors and promising clinical effects. Drug Deliv. Syst..

[71] Black K.L., Yin D., Ong J.M., Hu J., Konda B.M., Wang X., Ko M.K., Bayan J.-A., Sacapano M.R., Espinoza A. (2008). PDE5 inhibitors enhance tumor permeability and efficacy of chemotherapy in a rat brain tumor model. Brain Res..

[72] Zhang P., Zhang Y., Ding X., Xiao C., Chen X. (2020). Enhanced nanoparticle accumulation by tumor-acidity-activatable release of sildenafil to induce vasodilation. Biomater. Sci..

[73] Sheffield-Moore M., Wiktorowicz J.E., Soman K.V., Danesi C.P., Kinsky M.P., Dillon E.L., Randolph K.M., Casperson S.L., Gore D.C., Horstman A.M. (2013). Sildenafil increases muscle protein synthesis and reduces muscle fatigue. Clin. Transl. Sci..

[74] Treon S.P., Tournilhac O., Branagan A.R., Hunter Z., Xu L., Hatjiharissi E., Santos D.D. (2004). Clinical Responses to Sildenafil in Waldenstrom’s Macroglobulinemia. Clin. Lymphoma.

[75] Danial C., Tichy A.L., Tariq U., Swetman G.L., Khuu P., Leung T.H., Benjamin L., Teng J., Vasanawala S.S., Lane A.T. (2014). An open-label study to evaluate sildenafil for the treatment of lymphatic malformations. J. Am. Acad. Dermatol..

[76] Poklepovic A., Qu Y., Dickinson M., Kontos M.C., Kmieciak M., Schultz E., Bandopadhyay D., Deng X., Kukreja R.C. (2018). Randomized study of doxorubicin-based chemotherapy regimens, with and without sildenafil, with analysis of intermediate cardiac markers. CardioOncology.

[77] Booth L., Roberts J.L., Rais R., Cutler R.E.J., Diala I., Lalani A.S., Hancock J.F., Poklepovic A., Dent P. (2019). Neratinib augments the lethality of [regorafenib + sildenafil]. J. Cell. Physiol..

